# The Potentially Positive Role of PRPs in Preventing Femoral Tunnel Widening in ACL Reconstruction Surgery Using Hamstrings: A Clinical Study in 51 Patients

**DOI:** 10.1155/2014/789317

**Published:** 2014-11-06

**Authors:** Konstantinos A. Starantzis, Dimitrios Mastrokalos, Dimitrios Koulalis, Olympia Papakonstantinou, Panayiotis N. Soucacos, Panayiotis J. Papagelopoulos

**Affiliations:** 1st Department of Orthopaedics, ATTIKON University General Hospital, University of Athens, 124 62 Athens, Greece

## Abstract

*Purpose*. In this study, the early and midterm clinical and radiological results of the anterior cruciate ligament (ACL) reconstruction surgery with or without the use of platelet rich plasma (PRP) focusing on the tunnel-widening phenomenon are evaluated. *Methods*. This is a double blind, prospective randomized study. 51 patients have completed the assigned protocol. Recruited individuals were divided into two groups: a group with and a group without the use of PRPs. Patients were assessed on the basis of MRI scans, which were performed early postoperatively and repeated at least one-year postoperatively. The diameter was measured at the entrance, at the bottom, and at the mid distance of the femoral tunnel. *Results*. Our study confirmed the existence of tunnel widening as a phenomenon. The morphology of the dilated tunnels was conical in both groups. There was a statistical significant difference in the mid distance of the tunnels between the two groups. This finding may support the role of a biologic response secondary to mechanical triggers. *Conclusions*. The use of RPRs in ACL reconstruction surgery remains a safe option that could potentially eliminate the biologic triggers of tunnel enlargement. The role of mechanical factors, however, remains important.

## 1. Introduction

Following a successful ligament reconstruction, what matters most both for the surgeon and for the patient is the fast and durable integration of the graft. This practically means a rapid and permanent return in patients' high-level activities. And if time is not the priority, the quality of the graft integration is what everybody asks for.

Tunnel widening or tunnel enlargement is a previously unrecognized phenomenon associated with ACL reconstruction, which is referred to in the literature over the last two decades [1–7]. The clinical relevance of the phenomenon has been questioned [1, 3, 6, 8] but there are studies that consider this an important complication [9] making revision surgery problematic [10–13].

Several studies, in the past, have evaluated the importance of the various types of grafts [14, 15], reconstruction or fixation methods [16–24], and rehabilitation protocols [25, 26] but only recently surgeons have started to use blood material to biologically enhance graft integration [12, 27–30]. This study compared the early and midterm clinical and radiological results of the Anterior Cruciate Ligament (ACL) reconstruction surgery with or without the use of platelet rich plasma (PRP) focusing on the tunnel-widening phenomenon. The purpose of the study was to investigate the role of PRP in preventing the radiological widening of the femoral tunnels and in providing a better clinical outcome following ACL surgery.

## 2. Methods and Material

This was a double blind, prospective randomized study. Inclusion criteria were an isolated ACL rupture. Exclusion criteria were revision surgery, associated meniscal injuries requiring repair or total meniscectomy, and chondral lesions other than grade 1 requiring microfracturing. All patients were treated operatively with ACL reconstruction using hamstrings tendons (Semitendinosus and Gracilis) as a quadrupled graft with distal femoral fixation. Patients assigned for the study had to give informed consent of participation, according to the protocol approved by the hospital's ethics committee. Between December 2007 and June 2010, 60 consecutive patients were recruited for this study. Five of them were lost during the follow-up. Two patients were excluded because of associated meniscal lesions that required repair intraoperatively. Two patients (one of each group) had an ACL rerupture following early participation in contact sports, against medical advice. 51 patients have completed the assigned protocol; 74.5% (38 of 51) were male and 25.5% (13 of 51) were female. Recruited individuals were divided into two groups as follows: (1) control group (CG): ACL reconstruction with hamstring tendons (Semitendinosus and Gracilis) as a quadrupled graft, using distal fixation in the femur (Crosspin Linvatec or Endobutton Linvatec) and tibial fixation with a biodegradable interference screw (Linvatec) plus bone bridge suture anchoring (30 patients assigned, 26 patients completed the protocol); (2) PRP group (PRPG), ACL reconstruction as above plus PRP (30 patients assigned, 25 patients completed the protocol). The patients were assigned to each group randomly by the coordinator of this study (Konstantinos A. Starantzis) until 30 patients were allocated to each group. Two senior surgeons performed all the reconstructions using the same standardized surgical technique and type of fixation. All patients followed the same rehabilitation protocol. Both patients and surgeons were blinded to the groups upon the completion of the protocol.

### 2.1. Technique

#### 2.1.1. Collection of Platelet Rich Plasma

65 mL of blood is taken from the patient during the induction to anesthesia. A technician was in charge of preparing the samples in the anesthetic room next to the operating room. For PRP collection, 5 mL of an anticlotting agent (ACD-A) was added to 55 mL of blood using the Biomet GPS III kit (Biomet, Warsaw, IN), to which an ultrafiltering system was added in order to obtain a higher platelet concentration. The sample was centrifuged for 15 minutes at 3200 rpm resulting in plasma, platelets, and rest of the blood separation within the kit. From this, approximately 6 mL of PRP was collected. For the control group a 6 mL blood sample was used instead. For thrombin collection, 10 mL of the patient's blood (after the sample clotted) was centrifuged at 200 revolutions per minute over the course of 5 minutes and the skim was collected. Ten parts of this skim were mixed with 1 part of CaCl_2_. Once the process was completed, the application system was set up.

#### 2.1.2. Application of the Blood Clot

**Figure 1:**
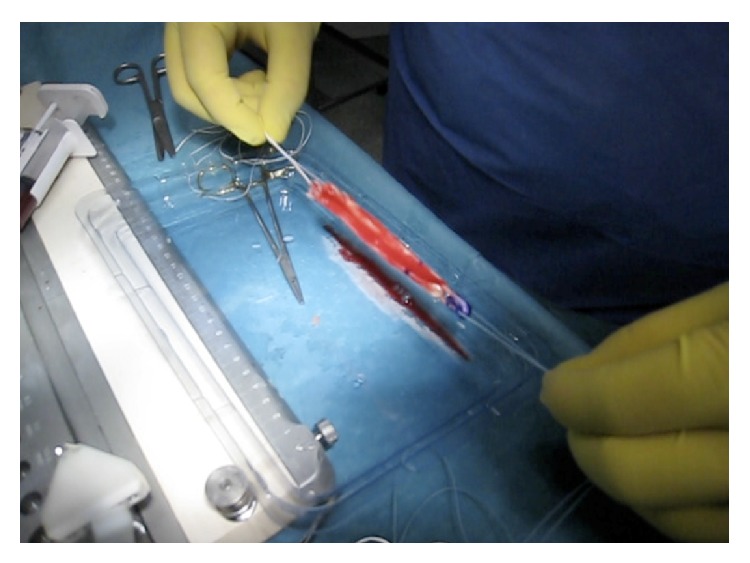
PRPs added between the strands of the graft and left to form a clot before the graft is pulled into the tunnel.

**Figure 2:**
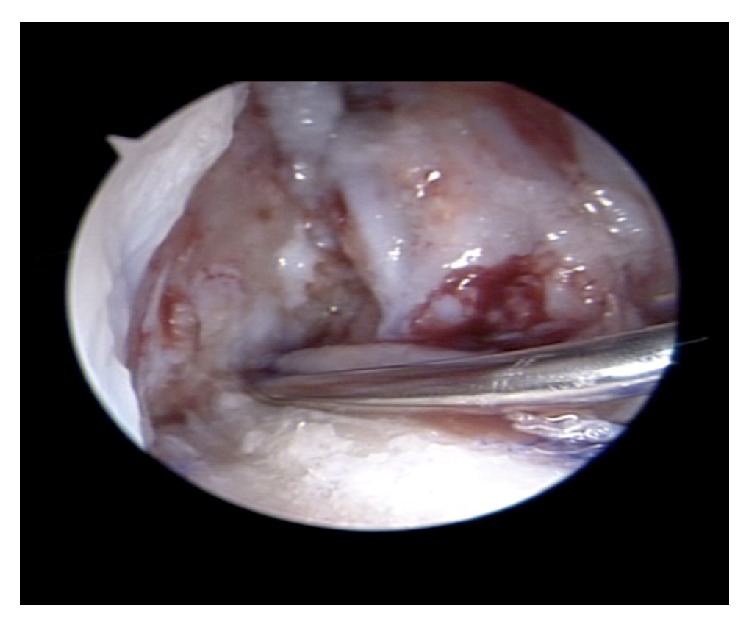
Once the graft is fixed within the tunnel, the remaining 3 mL of PRP is injected into the femoral tunnel using an introducer.

Half of the PRP (or the placebo sample) was added between the strands of the graft and left to form a clot before the graft was pulled into the tunnel (Figure 1). The graft was then inserted along the bone tunnels. Once it was fixed, the remaining 3 mL was injected into the femoral tunnel using an introducer (Figure 2). During and after the graft insertion and fixation the water pump was turned off to avoid dilution of the PRP.

### 2.2. Imaging Assessment

Patients were assessed on the basis of MRI scans. All MR examinations were performed on a 1.5 Tesla MR imaging unit (Philips NT Intera) using a dedicated knee coil. In all patients, coronal and axial T1-weighted images (TR/TE: 480–550/12–15 ms), sagital proton density/T2 weighted images (Tr; 2500–2700/TE: 20/90 ms), and coronal STIR (TR: 2900–3600/TE: 50 ms), or coronal proton density-weighted images with spectral fat saturation (TR: 3500–4100/TE: 15–20 ms), were obtained. Subsequently, T1-weighted images with spectral fat saturation after administration of intravenous gadolinium (T1W-FS; Dotarem 337 mg/mL, Gerbet) in a standard dose (0.1 mmol/kg) were obtained parallel to the femoral tunnel. Twenty-two MRI scans were performed on a different 1.5-Tesla MR unit with similar sequences and parameters. The MRI scans were performed early postoperatively (6 days min., 109 days max., and 46 days on average) and repeated at least one year postoperatively (11.2 months min., 37 months max., and 14.2 months on average).

**Figure 3:**
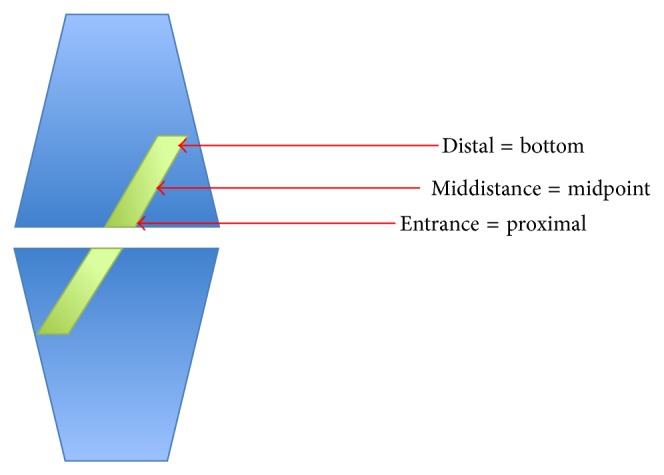
Schematic definition of different sites of measurements within the femoral tunnelin this study.

**Figure 4:**
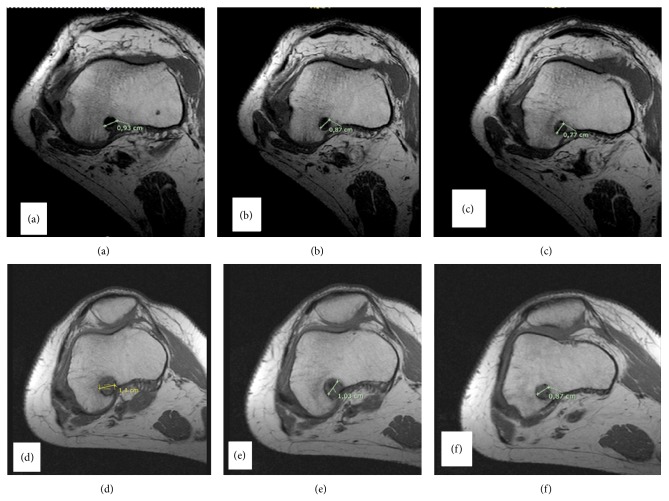
Comparison of femoral tunnel's axial MRI cuts performed on the same patient 1 and 12 months following ACL reconstruction. (a) Proximal 1 month, (b) middistance 1 month, (c) distal 1 month, (d) proximal 12 months, (e) middistance 12 months, and (f) distal 12 months.

An experienced musculoskeletal radiologist (Olympia Papakonstantinou), with no access to the clinical information, recorded, on two different occasions, the bone tunnel enlargement (by measuring the transverse diameters perpendicularly to the axis of the femoral tunnel). The diameters were measured at the entrance, at the bottom, and at the middistance of the femoral tunnel (Figure 3). The measurements were digitally obtained perpendicularly to the long axis of the tunnel (Figure 4). The highest widening measurement was recorded for each of the segments.

Additional CT scans were obtained in 12 volunteers in order to evaluate and validate the MRI measurements.

### 2.3. Clinical Assessment

Clinical assessment was performed preoperatively and 1 year postoperatively. In all patients, functional evaluations using the Lysholm score and Tegner scale were performed preoperatively and repeated in the annual follow-up visit. A Rolimeter assessment was performed to quantify the anterior draw shift preoperatively, immediately postoperatively, and 12 months following the surgery. A pivot shift test was performed and recorded in every clinical assessment. Evaluations were carried out by the senior surgeons (Dimitrios Mastrokalos and Dimitrios Koulalis), who were blinded to the groups, as aforementioned.

### 2.4. Data Analysis

An electronic database (FileMaker Pro Advanced 9.0v3) was created to securely record the data and the findings by the coordinator of this study (Konstantinos A. Starantzis).

A priori sample power analysis has demonstrated that a sample size of 25 evaluable patients per group was required to achieve a 90% probability of demonstrating a difference of more than 10% (SD: 10) between the two groups in % mean change of tunnel diameter with a significance of <5% (two tailed test).

Data was expressed as mean ± standard deviation (S.D.) or median (IQR) (in case of violation of normality) for continuous variables and as percentages for categorical data. The Kolmogorov-Smirnov test was utilized for normality analysis of the parameters. The comparison of variables at each time point was performed using the independent samples *t*-test or the Mann-Whitney* U* test in case of violation of normality. One factor Repeated Measures ANOVA model was used for the comparison of different time measurement of variables for each group. Pairwise multiple comparisons were performed using the method of Tukey critical difference. To indicate the trend in the first 12 months of intervention, the median percentage changes after 1 and 12 months, respectively, were calculated. Comparison of percentage change from baseline of parameters during the observation period between two groups was analyzed using the Mann-Whitney* U* test because of violation of normality. All tests were two-sided; statistical significance was set at *P* < 0.05. All analyses were carried out using the statistical package SPSS v 16.0 (Statistical Package for the Social Sciences, SPSS Inc., Chicago, Ill., USA).

## 3. Results

Mean age in the treated group was 29.4 ± 7.3 while in the control group it was 31.3 ± 8.0 (*P* > 0.05). Baseline tunnel diameter was considered to be equal with the drill diameter used intraoperatively for the tunnel preparation.

The mean tunnel diameter between the 2 groups during the observation period was not significantly different. There was a significant difference of the tunnel diameter for the PRPG (82.40 ± 4.59 to 85.79 ± 6.80) and the CG (80.19 ± 5.91 to 86.50 ± 8.88), respectively (*P* < 0.005), between the baseline and the 12-month assessment. Pairwise comparisons showed significant difference between the baseline and 12th month measurements (*P* < 0.05) for the PRPG and between baseline and 1st and 12th month (*P* = 0.05 and *P* < 0.0005), respectively, for the CG. There was no significant difference between the 2 groups of the percentile change of tunnel diameter from baseline to 1st and 12th month, respectively (*P* > 0.05). However, there was a statistical tendency towards a smaller percentile dilation of the mean tunnel diameter in the treated group at the annual follow-up (*P* = 0.062) (Table 1).

Further analysis of the raw data with regard to the tunnel diameter (proximal, in the middistance, and distal to the tunnel entrance) at baseline, 1st, and 12th month postoperatively demonstrated that there was a significant difference (*P* = 0.027) of the tunnel dilation, as a percentage of the drilled diameter, at the middistance of the tunnels in the annual follow-up between the PRPG and the CG: 2.50 mm (IQR 6.2) and 7.7 mm (IQR 15.2), respectively. No such difference was detected for the percentile dilation of the tunnel at the entrance and at the distal end during the follow-up (Table 3).

**Figure 5:**
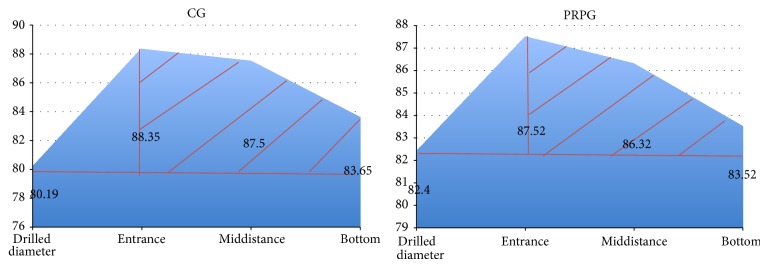
CG mean tunnel dilation at the entrance, middistance, and bottom end of the tunnel at 12 months. Horizontal red line shows the mean drilled diameter and shadowed area demonstrates the shape of tunnel dilation (conical). PRPG mean tunnel dilation at the entrance, middistance, and bottom end of the tunnel at 12 months. Horizontal red line shows the mean drilled diameter and shadowed area demonstrates the shape of tunnel dilation (conical).

Finally, the mean tunnel diameter at one year was larger in the entrance of the tunnels compared to the middistance, with the latter being subsequently larger compared to the diameter at the apex. This finding was constantly present in both groups. (Figure 5) and there was no significant difference between the 2 groups with regard to the tunnel diameters in these 3 areas from baseline to the 12th month's assessment (Table 4).

No significant difference of the Lysholm scores between the 2 groups during the observation period was detected. Pairwise comparisons showed significant difference between the preoperative and 12th month assessment (*P* < 0.0005) for both groups. There was no significant difference between the 2 groups of the mean percentile improvement of Lysholm scores at the 12th month's assessment, respectively (*P* = 0.434) (Table 2).

Rolimeter measurements improved significantly in both groups postoperatively to be 3.72 ± 0.54 from 10.12 ± 0.33 (*P* < 0.0005) for PRPG and 3.69 ± 0.74 from 10.19 ± 0.57 (*P* < 0.0005) for the control group. However, no difference was detected in between the two groups at the annual follow-up (*P* = 0.686) (Table 2). Pivot shift tests were negative in all patients postoperatively.

## 4. Discussion

The understanding of a biological phenomenon is essential prior to any attempt for intervention. Over the last two decades several studies have been published trying to figure out the graft healing procedure mainly in the ACL reconstruction surgery. Early animal studies have demonstrated that the bone-graft interface is initially characterized by increased numbers of fibroblasts and inflammatory cells, which are then replaced with progressively matured collagen fibers [31–35]. There is a literature consensus that the graft heals in three phases inside the tunnel through three major histological changes, namely, the maturation of fibrous tissue, the new bone formation, and the bone remodeling.

Kohno et al. [36], in their histological and immunohistochemical rabbit study, demonstrated that an empty space between the tendon graft and bone is observed at 1 week after surgery, while at 3 weeks granulation tissue composed of fibroblasts and small vessels occupy the interface space. In the same study it was concluded that FGF-2 and VEGF contribute to fibrous integration between the tendon and bone during the early postoperative stage and that BMP-2 and BMP-7 are specifically involved in bone remodeling leading to osseous integration. Reviewing the relevant literature it becomes obvious that growth factors have a number of crucial roles in bone-tendon healing [31, 37–47]. Whilst a large amount of information on these molecules has been produced over the last few years and decades, much work still needs to be done to fully understand their varied functions and multiple synergies.

Tunnel widening is a poorly understood phenomenon with many hypothetical etiologies that fall into two major categories, namely, the mechanical and the biological. Mechanical theories concentrate on the motion of the graft within the tunnel and the resultant wear. More biological theories rely on the inflammatory activity of the bone-graft interzone, which may account for a negative bone turnover locally.

Several studies, in the past, have evaluated the importance of the various types of grafts [14, 15], reconstruction or fixation methods [16–24], and rehabilitation protocols [25, 26] but only recently surgeons have started to use blood material to biologically enhance graft integration [12, 27–30].

### 4.1. The Site of Graft Fixation (the “Bungee Effect” and the “Windshield-Wiper Effect”)

Early studies have examined how the site of graft fixation affects the early motion of the graft within the bone tunnel and have tried to correlate this to certain patterns of tunnel widening (cone, cavital, and linear) [22, 48, 49]. Höher et al. [7] have reported that a suspensory graft fixation resulted in significantly greater longitudinal graft motion, the so called “bungee effect”, while an aperture fixation correlates with transverse graft motion known as the “windshield-wiper effect.” Rodeo et al. [50] showed that there is greater transverse graft motion as the distance from the fixation points increases, with the maximum of transverse motion at the tunnel aperture compared to the distal end of the tunnel. Many studies have confirmed the higher incidence of tunnel widening when extracortical fixation is used compared to aperture anatomical fixation [15, 16, 22]. A significant correlation (*P* < 0.01) was found between the incidence of tibial tunnel widening and the distance between the tip of the interference screw and the joint line as well [19]. The closer the joint to the tip of the screw, the lower the incidence of tunnel widening.

### 4.2. The Aggressive Rehabilitation Program

Hantes et al. demonstrated that a restricted rehabilitation program, in terms of delayed motion exercises, results in significantly decreased tunnel enlargement [26]. Vadalà et al. confirmed with the use of CT scan that early, aggressive, brace-free rehabilitation is associated with increased bone tunnel widening [25].

### 4.3. The Material of the Fixation Device

Tunnel widening is an issue with metal interference screws but remains a problem when polylactic-theoretically bioabsorbable screws are used [23]. However, polylactate/hydroxyapatite interference screws have demonstrated a reduced incidence of tunnel enlargement when used [23, 24, 51].

### 4.4. The Type of the Graft

In the early reports, this phenomenon was associated with allograft reconstruction [1–3]. More recent studies have failed to demonstrate a difference between allografts and autografts [11, 52]. Clatworthy et al. reported a significantly higher incidence of the phenomenon when hamstring autografts were used instead of patellar tendon autografts (BPTB). A multifactorial etiology was proposed involving the graft-bone interface, the stability of the fixation, and the micromotion in the healing zone causing inflammatory response that jeopardizes the quality of the graft integration within the bone [6]. L'Insalata et al. reported similar findings [52]. Webster et al. [53] reported in an RCT that 11% of the subjects who underwent ACL reconstruction with a BPTB graft had tunnel widening greater than 25%, compared to 94% of subjects who received a hamstring graft.

It seems that tendon-bone interface (SGT) is by definition a “slower” and biologically inferior interface for integration compared to the bone-to-bone interface (BPTB). Moreover hamstring autografts favor distal fixation methods that have been theoretically associated with increased motion of the graft within the tunnel, as aforementioned.

*Biological Factors* have also been involved in tunnel enlargement. Cytokine-mediated nonspecific inflammatory response, foreign body immune reaction, and products' toxicity within the tunnel have been involved in the biological explanations of the phenomenon [7, 11]. After the graft placement and fixation, granulation tissue develops in the graft-tunnel interface creating a biologically active zone to facilitate graft integration. Various mediators are released into this zone that could potentially stimulate bone absorption along the tunnel walls [6, 7, 52]. Drilling and its thermogenic effect may also cause localized bone necrosis [1]. Moreover, the graft itself can also cause an inflammatory response due to the cell necrosis that will occur [54]. Localized inflammation triggers the release of cytokines and, as a result of this, a negative bone turnover locally that can lead to the tunnel enlargement.

In [7] Silva et al. highlighted the importance of a possible biological activity within the tunnel over mechanical factors as the most likely explanation for the enlargement of the tunnels at the middle, rather than at the entrance or the bottom [12]. However, Höher et al. [7] adopted a theoretical concept for the pathogenesis of bone tunnel enlargement which combined mechanical and biological triggers that can cause a local biological response and bone resorption as a result of this.

Our study confirmed the existence of tunnel widening as a phenomenon in ACL reconstruction using hamstrings tendons. This comes in line with most of the published papers, adding significance to our results. The use of PRPs has failed to significantly reduce the magnitude of the phenomenon in the entrance of the tunnel. However, there was a statistical significant difference in the midpoint of the tunnels between the two groups with regard to the tunnel enlargement as a percentage of the drilled diameter. This finding, which is, to the best of our knowledge, reported for the first time in the literature, may be suggestive of the potential role of the biological reaction secondary to the mechanical triggers. The fact that PRPs have little or no effect in preserving tunnel diameters in the proximal part of the tunnels where graft motion is maximum may suggest that mechanical triggers of inflammation can only be modified (suppressed) within certain limits of graft motion. The minimal or no tunnel enlargement at the tunnel aperture in both groups is supportive of the seminal role of the mechanical factors in the pathogenesis of the phenomenon.

Another important finding of this study was that the morphology of the dilated tunnels in one year's time following reconstruction is conical in both groups. Conical dilation of the tunnels is best seen, schematically, in Figure 5 and it applied for both groups. In our opinion the conical shape of the enlargement is supportive of the mechanical theory involved in the pathogenesis of the phenomenon. The rationale for this is that the magnitude of the dilation is directly related to the amount of graft motion inside the tunnel; as with distal fixation methods you anticipate the maximum movement of the graft around the tunnel entrance and less movement at the bottom end of the tunnel (windshield wiper phenomenon).

An interesting imaging finding is that in at least two-thirds of the tunnels in the treated group a tunnel diameter of less than 10 mm was preserved in the long term radiological follow-up. The clinical relevance of this finding is that a well-preserved tunnel—as in the PRP group—could potentially give more (and easier) surgical options for one-stage revision compared to the nontreated group.

Our study also confirmed that there is no clinical relevance of the phenomenon with regard to the clinical tests and the self-assessment scores in the early and midterm follow-up period, as early studies have reported so far.

Platelet rich plasma can be categorized as autografts and as such carries of similar risks and potential complications. In our opinion the risk of infection is realistically the most significant amongst all risks. These in theory include, but are not limited to, the risk of an uncontrolled inflammatory response, which could potentially lead to arthrofibrosis or graft rupture. Conditions that could potentially be considered as contraindications for the use of PRP in ACL reconstruction surgery include hematologic dyscrasias with platelet dysfunction, bacteremia, and malignancy. As for the safety of PRPs, this study has not demonstrated an increase rate of complications in the treated group. More specifically, there were 2 graft reruptures—one for each group following early participation in sports. No infection was documented in either group.

Strong points of our study include the methodology and the homogeneity of the sample, of the fixation method, and the rehabilitation protocol as well as the high intraobserver agreement in the tunnel measurements. Weak points are the marginal power of the sample and the use of MRI instead of CT scan to measure the tunnel diameters. The power of the sample has been reduced from strong to marginal, firstly because the difference of tunnel diameter within the two groups was less than 10% and, secondly, because of those patients lost during the follow-up. The use of CT scan for scientific purposes carries an ethical issue regarding the radiation exposure of the patients. We have only performed 12 crosschecks in 10 volunteers who have given their written consent after explaining to them the risks of participating and the objectives of our survey.

One could argue whether an inherent limitation of the study was the method of PRP application. Murray in an in vitro study has suggested that “the use of low concentrations of thrombin (10.5 IU/mL) may be beneficial in applications where a faster set time and enhanced cell migration are desirable and the gel mechanical strength is of secondary importance” [42]. Thrombin and various types of scaffolds have been advocated to increase the time PRPs are maintained within the area of interest and reduce the dilution of the important growth factors in the articular fluid [55, 56]; however, comparative studies do not exist to support a potentially superior effect of any of these methods. Nonetheless this study assesses the efficacy of the previously described method of PRPs' application.

## 5. Conclusions

This study has confirmed femoral tunnel widening as a phenomenon in ACL reconstruction surgery using hamstrings and distal fixation of the graft. PRPs did not result in a better or faster recovery, clinical outcome, or—most importantly—a reduction in the revision rate. PRPs did have a significant effect on minimizing the dilation in the middistance of the tunnels, which could potentially be beneficial in the case of a revision reconstruction. This has not been confirmed statistically in this study because the size of the sample was not powerful enough to detect or to exclude such difference.

In conclusion, the present results suggest that PRPs in ACL surgery may assist in decreasing tunnel enlargement and deserve further clinical evaluation.

## Figures and Tables

**Table 1:** Comparison of mean tunnel diameter between groups during the observation period.

	PRPG	CG	*P* value between groups
	Mean ± SD	Mean ± SD	
Drilled diameter	82.40 ± 4.59	80.19 ± 5.91^#^	0.144
1st month diameter	83.33 ± 4.67^*^	80.88 ± 6.39^#^	0.135
12th month Diameter	85.79 ± 6.80	86.50 ± 8.88	0.749
*P* value within group	**P** = **0.005**	**P** < **0.0005**	

	Median (IQR)	Median (IQR)	

% change from drilled 1st month	0.78 (1.6)	0.00 (1.2)	0.693
% change from drilled 12th month	2.96 (5.2)	6.87 (11.8)	**0.062**

^*^ *P* > 0.05 versus baseline, ^#^ *P* < 0.005 versus 12th month.

**Table 2:** Comparison of Lysholm and Rolimeter variables between groups during the observation period.

		PRPG	CG	*P* value between groups
Lysholm	Preoperative	52.00 ± 11.89	54.15 ± 12.59	0.533
	Postoperative	96.36 ± 43.04	95.08 ± 63.20	0.149
	*P* value within group	**P** <** 0.0005**	**P** <** 0.0005**	
	% change pre- and postoperatively	90.0 (40.7)	81.7 (71.5)	0.434

Rolimeter	Preoperative	10.12 ± 0.33	10.19 ± 0.57	0.583
	Postoperative	3.72 ± 0.54	3.69 ± 0.74	0.879
	*P* value within group	**P** <** 0.0005**	**P** <** 0.0005**	
	% change pre- and postoperatively	−60.0 (10.0)	−60.0 (10.0)	0.686

All values are presented as mean ± SD.

% change pre- and postoperatively are presented as median (IQR).

**Table 3:** Comparison of proximal, middistance, and distal tunnel mean diameters between groups during the observation period.

		PRPG	CG	*P* value between groups
Proximal	Drilled diameter	82.40 ± 4.59^#^	80.19 ± 5.91^#^	0.144
	1st month diameter	83.64 ± 5.11^#^	81.19 ± 6.43^#^	0.140
	12th month diameter	87.52 ± 8.26	88.35 ± 11.43	0.769
	*P* value within group	**P** = **0.001**	**P** = **0.0005**	
	% change from drilled 1st month	0.0 (2.5)	5.0 (8.8)	0.910
	% change from drilled 12th month	0.0 (2.5)	6.9 (11.9)	0.257

Middistance	Drilled diameter	82.40 ± 4.59	80.19 ± 5.91^#^	0.144
	1st month diameter	83.32 ± 4.63	80.81 ± 6.41^#^	0.116
	12th month diameter	86.32 ± 7.75^*^	87.50 ± 9.63	0.633
	*P* value within group	**P** = **0.005**	**P** = **0.0005**	
	% change from drilled 1st month	0.0 (2.4)	0.0 (0.3)	0.792
	% change from drilled 12th month	2.50 (6.2)	7.7 (15.2)	**0.027**

Distal	Drilled diameter	82.40 ± 4.59	80.19 ± 5.91^#^	0.144
	1st month diameter	83.04 ± 4.67	80.65 ± 6.39^#^	0.135
	12th month diameter	83.52 ± 6.46	83.65 ± 8.00	0.948
	*P* value within group	*P* = 0.473	**P** <** 0.0005**	
	% change from drilled 1st month	0.0 (0.0)	0.0 (3.7)	0.821
	% change from drilled 12th month	0.0 (0.0)	3.6 (10.0)	0.163

^*^ *P* < 0.05 versus baseline, ^#^ *P* < 0.005 versus 12th month.

**Table 4:** Comparison of tunnel diameter between groups at different locations one year postoperatively.

	Tunnel diameter (mm)	*P* value
	Drilled	
PRPG	82.40 ± 4.59	0.144
CG	80.19 ± 5.91	

Proximal		
PRPG	87.52 ± 8.26	0.769
CG	88.35 ± 11.43	

Mid-distance		
PRPG	86.32 ± 7.75	0.633
CG	87.50 ± 9.62	

Distal		
PRPG	83.52 ± 6.46	0.948
CG	83.65 ± 8.00	
